# Two novel low-power and high-speed dynamic carbon nanotube full-adder cells

**DOI:** 10.1186/1556-276X-6-519

**Published:** 2011-09-02

**Authors:** Mehdi Bagherizadeh, Mohammad Eshghi

**Affiliations:** 1 Science and Research Branch of Islamic Azad University, Tehran, Iran; 2 Shahid Beheshti University, GC, Tehran, Iran

**Keywords:** carbon nanotube transistor, dynamic full adder, low power, high speed

## Abstract

In this paper, two novel low-power and high-speed carbon nanotube full-adder cells in dynamic logic style are presented. Carbon nanotube field-effect transistors (CNFETs) are efficient in designing a high performance circuit. To design our full-adder cells, CNFETs with three different threshold voltages (low threshold, normal threshold, and high threshold) are used. First design generates *SUM *and *COUT *through separate transistors, and second design is a multi-output dynamic full adder. Proposed full adders are simulated using HSPICE based on CNFET model with 0.9 V supply voltages. Simulation result shows that the proposed designs consume less power and have low power-delay product compared to other CNFET-based full-adder cells.

## Introduction

Carbon nanotube field-effect transistors (CNFETs) are one of the new devices for designing low-power and high-performance circuits [1,2]. Scaling of complementary metal-oxide semiconductor (CMOS) technology to the nano ranges has many limitations and leads to increase the leakage currents, power dissipation, and short-channel effects [1-3]. CNFET technology mitigates these problems and these limitations of CMOS technology. Carbon nanotubes (CNTs) are sheets of graphite which formed into cylinders. A nanotube with one layer of carbon atoms is single-wall carbon nanotube (SWCNT), and a CNT with multiple layers of carbon atoms is multi-wall carbon nanotube (MWCNT). SWCNT has the ability to act as a conductor (metal) and as a semiconductor as well [2,4].

The threshold voltage of a CNFET depends to its size, Equation 1:

(1) $$V_{\text{th}}=\frac{\sqrt{3}}{3}\frac{aV_{\pi }}{eD_{\text{CNT}}}$$

Where *e *is the unit electron charge, *V*_π _= 0.033 eV is the carbon π-π bond energy, *a *= 2.49 Å (angstrom) is the carbon to carbon atom distance, and *D*_CNT _is the CNT diameter, Equation 2:

(2) $${\text{D}}_{\text{CNT}}=\frac{\text{α}\sqrt{3}}{\text{π}}\sqrt{{\text{n}}^{2}+{\text{m}}^{2}+\text{nm}}$$

In Equation 2, *n *and *m *are chirality of CNT and *α *= 0.142 nm is the inter-atomic distance between each carbon atom and its neighbor [1,2,5].

As indicated in Equation 1, the threshold voltage of CNFETs depends to the inverse of the diameter of nanotube used as a channel. As a result, different transistors with different turn on voltage can be implemented by changing diameter of CNT [1-3,6].

A full adder is one of the most significant parts of a processor. In all the arithmetic operations such as division, multiplication, and subtraction, full adders are used as essential components. The full adder also is the core element of complex arithmetic circuits. As a result, increasing the performance of a full adder leads to increase the performance of the whole system [4,6-15].

There are many implementations of full adders which are implemented using metal-oxide-semiconductor field-effect transistor (MOSFET) and CNFET technologies. These full adders are in standard static logic and in dynamic logic. Dynamic logic style has some advantages compared to the static logic style. These advantages are as follows: the number of transistors is low, these transistors do not have any static power consumption, the speeds of switching are high, and the voltage levels are full swing. Dynamic logic style has also disadvantage of high switching activity [10].

In this paper, we present two novel carbon nanotube full-adder cells in dynamic logic style. These proposed full adders are simulated using HSPICE based on CNFET model with 0.9 V supply voltage. Simulation result shows that the proposed designs consume less power and have low power-delay product (PDP) compared to other classical CMOS and CNFET-based full-adder cells, presented in other papers.

The rest of this paper is organized as follows: "Literature review on full-adder cells in MOSFET and CNFET technologies" presents some full adders which are designed using MOSFET and CNFET technologies. In "Proposed full adder cell designs," we introduce two novel high-speed and low-power carbon nanotube full adders in dynamic logic. "Simulation results and comparison" compares the proposed designs with other designs. "Conclusion" concludes the paper.

### Literature review on full-adder cells in MOSFET and CNFET technologies

There are different implementations of full-adder cells which have been proposed in many researches [4,6-15]. In this section, some of these full adders which are implemented using MOSFET and CNFET technologies are introduced.

The complementary CMOS (C-CMOS) full adder [7] has 28 transistors and composed of p-channel MOS (PMOS) transistors as a pull-up network and n-channel MOS (NMOS) transistors as a pull-down network. The voltage levels of this full adder are full swing, but the number of transistors of this full adder is high.

The complementary pass-transistor logic full adder [5] has 32 transistors, and the speed of switching of this design is high. It has full swing voltage levels. Transmission-gates CMOS full adder [12] has 20 transistors. It is composed of a PMOS transistor and an NMOS transistor in a parallel form. The multi-output dynamic full adder [10] has 21 transistors, 15 transistors to product SUM and $\bar{\text{COUT}}$ outputs, and 6 transistors to invert inputs. The 26T full-adder cell [12] is composed of 10 transistors to produce XOR and XNOR functions in the first stage and 16 transistors to create COUT and SUM outputs in the second stage.

The carbon nanotube full adder which is implemented by means of majority function is presented in [6]. In this design, a three-input majority function is used to implement COUT and a five-input majority function is used to implement SUM, as presented in Equation 3. "Majority" function is an odd-inputs logic circuit that performs as a majority voter to determine the output of the circuit:

(3) $$\text{SUM}=\text{Majority}\left(\text{A},\text{B},\text{C},\bar{\text{COUT}},\bar{\text{COUT}}\right)$$

In [14], another carbon nanotube full adder based on majority function is presented which is a low-voltage and energy-efficient design. This full adder is composed of eight transistors and five capacitors.

A high-speed capacitor-inverter-based carbon nanotube full adder based on majority-not function is presented in [13]. To design this full adder, NAND and NOR functions are used. The output SUM of this full adder is implemented by Equation 4:

(4) $$\text{SUM}=\text{Minority}\left(\text{A},\text{B},\text{C},2*\text{NAND}\left(\text{A},\text{B},\text{C}\right),2*\text{NOR}\left(\text{A},\text{B},\text{C}\right)\right)$$

The carbon nanotube full adder presented in [15] is another majority function based with 14 transistors and 3 capacitors. To design this full adder, NAND and NOR functions are also used.

### Proposed full-adder cell designs

Our proposed full-adder cells are in dynamic logic style. There are two phases in a dynamic logic, pre-charge phase and evaluation phase. The pre-charge phase is accrued when Clock = 0; otherwise, the circuit enters the evaluation phase. A PMOS transistor connects the output nodes to their Vdd, at pre-charge phase. To avoid incorrect functionality and charge sharing problem, all the input values should be changed at pre-charge phase. In our designs, three capacitors and CNFETs with three different threshold voltages, low threshold, normal threshold, and high threshold, are used.

### Proposed low-power dynamic carbon nanotube full adder

**Figure 1:**
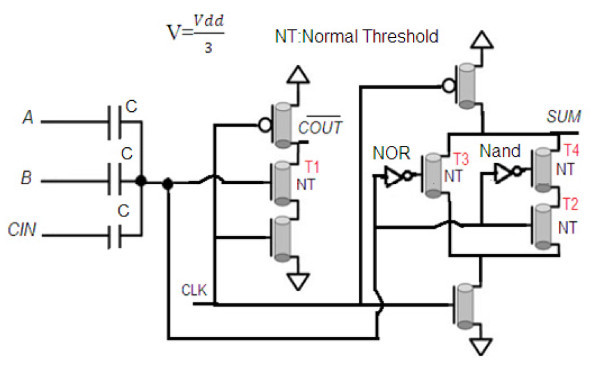
**Primary schema for the proposed low power dynamic carbon nanotube full adder**.

The truth table of a full adder is shown in Table 1. As indicated in this table, SUM output is "1" if the sum of three inputs (SIGMA) is equal to "1" or "3"; otherwise, it is equal to "0." COUT output is equal to "1" if SIGMA is equal to "2" or "3"; otherwise it is equal to "0." The simplified truth table of a full adder is shown in Table 2. Based on these tables, our full adder is designed. Figure 1 shows primary schema for the proposed low-power dynamic carbon nanotube full adder (first design).

**Table 1:** Truth table of a full adder

A	B	CIN	COUT	SUM
0	0	0	0	0
0	0	1	0	1
0	1	0	0	1
0	1	1	1	0
1	0	0	0	1
1	0	1	1	0
1	1	0	1	0
1	1	1	1	1

**Table 2:** Simplified truth table of a full adder

SIGMA	COUT	SUM
0	0	0
1	0	1
2	1	0
3	1	1

In this design, the T1, T2, T3, and T4 transistors are NMOS transistors with normal thresholds. The NOR and NAND gates contains an NMOS transistor with Vt = *vt *and a PMOS with Vt = Vdd - *vt*. In a NOR gate, when all of the three inputs (A, B, C) are "0," this output is equal to "1"; otherwise, in all of the other minterms, this output is equal to "0." In a NAND gate, when all of the inputs are "1," this output is equal to "0"; otherwise, in all of the other minterms, this output is equal to "1."

**Figure 2:**
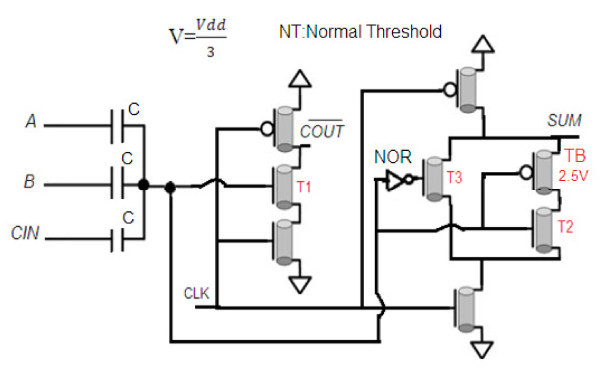
**Final schema for the proposed low power dynamic carbon nanotube full adder**.

Figure 2 shows the final schema for the proposed low-power dynamic carbon nanotube full adder. As shown in this figure, to obtain more efficiency and enhancing the proposed design, we eliminate NAND gate and replace the NMOS T4 transistor with a PMOS transistor (TB) with high threshold, Vt = 2.5*v*, where $v=\frac{\text{Vdd}}{3}$. When all of the inputs are "1," this transistor is "off"; otherwise, it is "on."

This design is evaluated in all minterms. When clock is equal to "0," the circuit enters the pre-charge phase. In this phase, a PMOS transistor connects the SUM and $\bar{\text{COUT}}$ outputs to their Vdd. At evaluation phase, clock is equal to "1." In this phase, when SIGMA is "0," T3 transistor is "on," and T1 transistor is "off," as a result SUM output is equal to "0" and $\bar{\text{COUT}}$ output is unchanged and it is equal to "1." At this phase when SIGMA is "1," the T1, T2, and T3 transistors are "off." As a result, both outputs, SUM and $\bar{\text{COUT}}$, are unchanged and they are equal to "1." When SIGMA is "2," then the T1, T2, and TB transistors are "on." As a result, both outputs are equal to "0." When SIGMA is "3," then T3 and T4 transistors are "off." As a result, SUM output is unchanged and it is equal to "1" and $\bar{\text{COUT}}$ output is equal to "0." Table 3 shows the state of all transistors for different values of SIGMA.

**Table 3:** State of transistors at evaluation phase for different values of SIGMA

SIGMA	T1	T2	T3	TB	SUM	$\bar{COUT}$
0	Off	Off	On	On	"0"	Unchanged ("1")
1	Off	Off	Off	On	Unchanged ("1")	Unchanged ("1")
2	On	On	Off	On	"0"	"0"
3	On	On	Off	Off	Unchanged ("1")	"0"

### Proposed multi-output dynamic carbon nanotube full adder

**Figure 3:**
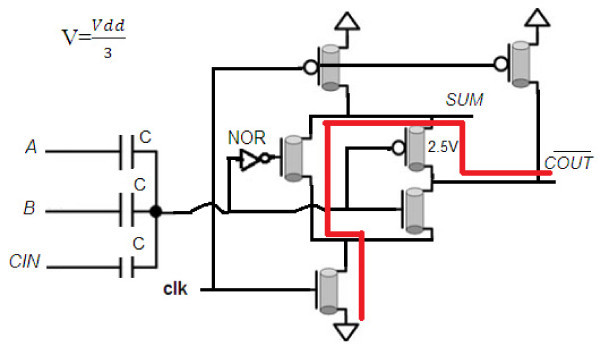
**Primary schema for the proposed multi-output dynamic full adder**.

Second design is a multi-output dynamic carbon nanotube full-adder cell. To design this full adder, three capacitors and nine CNFETs are used. The primary schema of this full adder is shown in Figure 3. In this design, two PMOS transistors are used to charge the outputs ($\bar{\text{COUT}}$, SUM) in pre-charge phase. In order to create $\bar{\text{COUT}}$ output, an NMOS normal threshold transistor is used. This transistor, along with two other transistors and a NOR gate, is used to create SUM output.

**Figure 4:**
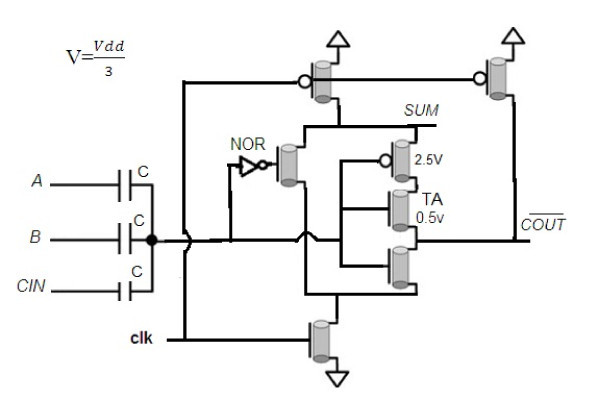
**Final schema for the proposed multi-output dynamic full adder**.

Figure 3 shows that when SIGMA is "0," then there is a path that connects the GND (= "0") to $\bar{\text{COUT}}$. To overcome this problem, an NMOS transistor (TA) with low threshold (Vt = 0.5*v*) is added to the circuit. Figure 4 shows this modification and final design of this multi-output dynamic full adder. In this circuit, when SIGMA = "0" this transistor is off and leads to disconnect the path from GND to $\bar{\text{COUT}}$.

## Simulation results and comparison

**Figure 5:**
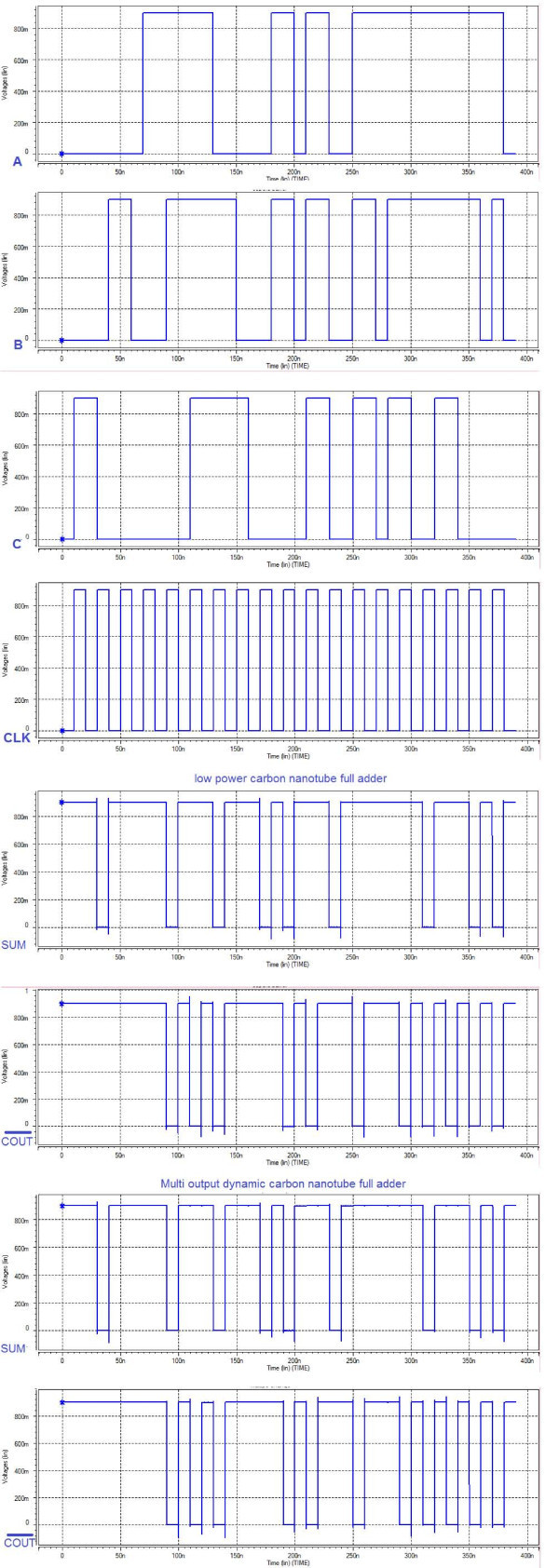
**Input and output signal for both proposed designs at 0.9 V supply voltage**.

Through a computer simulation, we compare our proposed full-adder cells to four other different exiting carbon nanotube designs [6,13-15]. HSPICE based on CNFET model [16,17] is used to simulate these full-adder cells. To compare these full adders, three criteria, delay, power dissipation, and power-delay product (PDP), are employed. The supply voltage is considered 0.9 V for all circuits. The delay is calculated from 50% of voltage level of input to 50% of voltage level of output. For being more realistic, we place buffers (two cascaded inverter) in the two outputs. The frequency of clock signal is 50 MHz. For both proposed full-adder cells, the input and output signals at the 0.9 V supply voltages are depicted in Figure 5.

The results of simulation for 0.9 V Vdd voltage are shown in Table 4. From delay point of view, among the existing full adders, the design in [15] is the fastest full adder and the design in [13] is the slowest full adder. Proposed low power dynamic carbon nanotube full adder is 46% slower than the design in [15], 12% slower than the design in [6], 39% slower than the design in [14], and 21% faster than the design in [13]. Among the existing full adders, the power consumption of our proposed low-power dynamic carbon nanotube full adder is lowest, and it is 48% less than the design in [15], 87% less than the design in [14], 75% less than the design in [13], and 89% less than the design in [6]. The PDP of the proposed full adder is 90% lower than the design in [6], 81% lower than the design in [13], 82% lower than the design in [14], and 3% lower than the design in [15].

**Table 4:** Comparison between the proposed designs and others CNT full adders

Full adders	Parameters		
	Delay (pS)	Power (μW)	PDP × E-17 (SW)
Design in [6]	78.3	1.05	8.20
Design in [13]	114	0.332	3.80
Design in [14]	53.6	0.783	4.20
Design in [15]	47.8	0.129	0.618
Proposed low-power dynamic CNT full adder	89.3	0.067	0.596
Proposed multi-output dynamic CNT full adder	84.3	0.062	0.519

Proposed multi-output dynamic full adder is 7% slower than the design in [6], 26% faster than the design in [13], 36% slower than the design in [14], and 43% slower than the design in [15]. This proposed full adder consumes 91% less power than the design in [6], 78% less than the design in [13], 90% less than the design in [14], and 50% less than the design in [15]. The PDP of our proposed multi-output dynamic full adder is 91% lower than the design in [6], 84% lower than the design in [13],85% lower than the design in [14], and 15% lower than the design in [15].

## Conclusion

In this paper, we proposed two novel low-power carbon nanotube dynamic full adders. Transistors with tree different threshold voltages, by changing diameter of CNT, were used to implement the proposed dynamic full adders. In the first proposed full adder, SUM and $\bar{\text{COUT}}$ were generated through separate transistors. Second proposed full adder, however, was a multi-output dynamic full adder. Simulation results showed that both proposed designs had less power consumption and low PDP, compared to the previous CNFET designs. Table 4 shows comparison between the proposed full-adder designs and circuits proposed in [6,13-15].

## Competing interests

The authors declare that they have no competing interests.

## Authors' contributions

MB designed and simulated the proposed circuit, as part of his Master of Science thesis research. ME was the advisor in his thesis research and gave the general idea in the research and also helped in critical drafting of manuscript and presentation of the results.
